# Supplementary material for: Prospective Comparison of Handheld Ultrasound Devices from Different Manufacturers with Respect to B-Scan Quality and Clinical Significance for Various Abdominal Sonography Questions
Source: Diagnostics (Basel). 2023 Dec 8;13(24):3622. doi: 10.3390/diagnostics13243622 (PMC10742722; doi:10.3390/diagnostics13243622)
Supplement: Supplementary file 1 [file diagnostics-13-03622-s001.zip › diagnostics-2642235-supplementary.pdf]

Supplementary Material

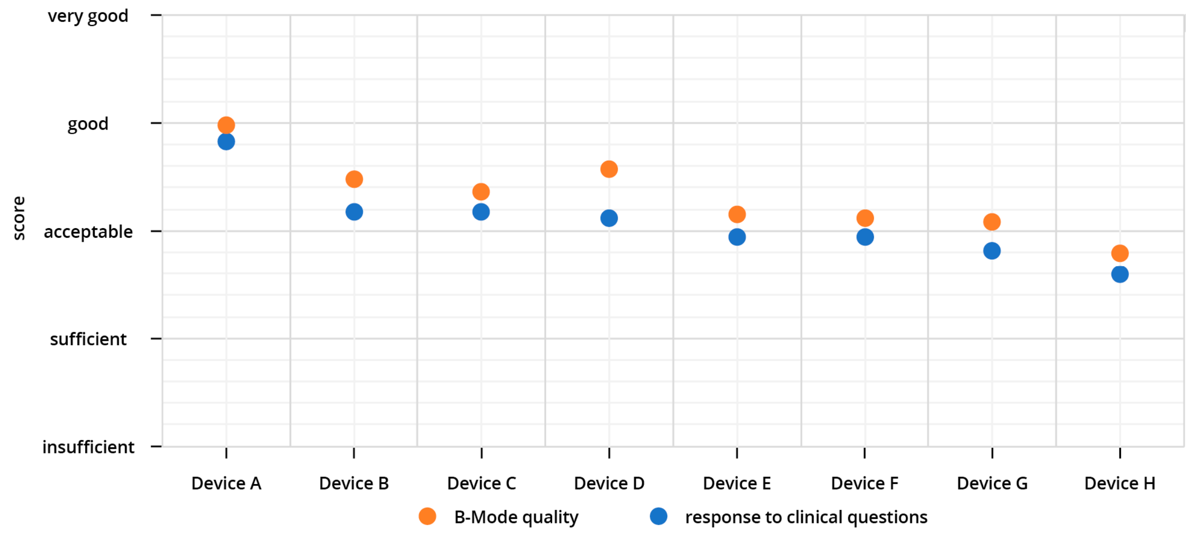

Supplement Figure S1: *Comparison of the scores obtained from both evaluations for each device used*

Supplement Table S1: *Results of the evaluated B-scan quality per evaluator.*

Examiner	Alpinion	Butterfly	Clarius	Echonous	Iviz	Lumify	Vscan Air	Youkey
1	3,00	3,71	3,00	3,14	3,29	3,29	**4,14**	3,86
2	2,57	**3,71**	2,86	3,43	2,71	2,71	**3,71**	3,29
3	2,86	2,71	2,57	3,29	2,57	3,00	**3,57**	2,86
4	2,43	2,86	2,43	2,86	2,57	2,43	**3,14**	2,86
5	2,71	3,43	2,29	3,00	3,14	3,00	**4,14**	3,29
6	2,29	3,00	2,14	3,00	2,57	2,43	**3,57**	3,14
7	1,43	2,14	1,86	**2,71**	2,57	2,14	**2,71**	1,86
8	2,71	2,86	2,14	3,43	3,14	3,14	**3,86**	3,00
9	2,57	4,14	2,86	4,29	3,86	3,57	**4,71**	3,43
10	3,00	3,29	3,00	3,29	2,71	3,29	**4,29**	3,29
11	2,14	2,29	2,00	2,43	2,29	2,43	**3,14**	2,43
12	2,86	3,43	2,43	3,29	3,00	2,86	**3,57**	3,29
13	2,71	2,86	2,43	3,14	2,86	3,00	**4,14**	3,57
14	3,71	4,00	3,00	4,14	3,86	3,86	**4,29**	4,29

Supplement Table S2*: Results of the evaluated clinical significance per evaluator.*

Examiner	Alpinion	Butterfly	Clarius	Echonous	Iviz	Lumify	Vscan Air	Youkey
1	2,86	3,86	3,43	3,86	3,57	3,43	**4,14**	3,71
2	2,57	**4,14**	3,29	3,71	2,71	2,86	**4,14**	3,57
3	3,29	3,43	3,29	3,71	3,00	2,86	**3,57**	3,14
4	2,71	3,14	2,71	3,14	3,29	3,14	**3,57**	3,14
5	2,57	3,57	2,71	3,00	3,00	2,86	**4,14**	3,29
6	2,71	3,29	2,71	**3,57**	2,71	2,57	**3,57**	3,29
7	1,43	2,14	2,14	2,71	2,57	2,14	2,71	1,86
8	3,29	3,29	3,43	4,00	3,86	3,57	**4,14**	3,86
9	2,29	3,86	3,14	4,43	3,71	3,57	**4,71**	3,43
10	3,29	4,00	3,71	**4,14**	3,00	3,71	**4,14**	3,71
11	2,43	2,71	2,86	**3,43**	2,29	2,86	3,29	2,86
12	3,71	3,71	3,43	3,71	3,57	3,43	**3,86**	3,57
13	2,86	3,43	3,14	3,71	3,14	3,00	**4,43**	3,57
14	3,71	4,00	3,14	4,14	3,57	3,57	**4,29**	4,00

Supplement Table S3: *P-values in the comparison of the B-scan quality of the individual HHUS devices.*

	DeviceA	Divice B	Device C	Device D	Device E	Divice F	Divice G
Device B	0.003						
Device C	0.003	0.81					
Device D	0.0004	0.74	0.58				
Device E	<0.0001	0.07	0.06	0.15			
Device F	<0.0001	0.08	0.07	0.15	0.98		
Device G	<0.0001	0.01	0.009	0.02	0.39	0.41	
Device H	<0.0001	<0.001	<0.001	0.004	0.01	0.013	0.11

Supplement Table S4: *P-values in the comparison of the clinical significance of the individual HHUS devices.*

	DeviceA	Divice B	Device C	Device D	Device E	Divice F	Divice G
Device B	<0.0001						
Device C	<0.0001	0.63					
Device D	0.002	0.55	0.31				
Device E	<0.0001	0.028	0.11	0.005			
Device F	<0.0001	0.018	0.078	0.003	0.89		
Device G	<0.0001	0.012	0.056	0.002	0.89	0.99	
Device H	<0.0001	0.018	<0.0001	<0.0001	0.02	0.028	0.028

Supplement Table S5: *P-values in the comparison of the B-scan quality of the different sonographic questions.*

	Gallbladder	Ascites	IVC	Needle in vitro	Pancreas	Hepatic Lesion
Ascites	< 0.001					
IVC	0.008	0.88				
Needle in vitro	0.012	0.42	0.99			
Pancreas	0.14	< 0.001	< 0.001	< 0.001		
Hepatic Lesion	0.41	< 0.001	0.001	0.002	0.77	
Needle in situ	0.78	< 0.001	0.008	0.009	0.37	0.99

Supplement Table S6: *P-values in the comparison of the clinical significance of the different sonographic questions*

	Gallbladder	Ascites	IVC	Needle in vitro	Pancreas	Hepatic Lesion
Ascites	< 0.001					
IVC	< 0.001	0.15				
Needle in vitro	< 0.001	0.26	0.81			
Pancreas	0.39	< 0.001	< 0.001	< 0.001		
Hepatic Lesion	0.92	< 0.001	< 0.001	< 0.001	0.37	
Needle in situ	0.88	< 0.001	< 0.001	< 0.001	0.46	0.83
